# How patient-reported outcomes and experience measures (PROMs and PREMs) are implemented in healthcare professional and patient organizations? An environmental scan

**DOI:** 10.1186/s41687-024-00795-9

**Published:** 2024-11-15

**Authors:** Véronique Lowry, Vanessa Tremblay-Vaillancourt, Priscilla Beaupré, Marie-Dominique Poirier, Marie-Ève Perron, Jessica Bernier, Anaëlle Morin, Caroline Cormier, Jeannie Haggerty, Sara Ahmed, Magaly Brodeur, Geneviève David, Sylvie Lambert, Maude Laberge, Diana Zidarov, Regina Visca, Thomas G. Poder, Hervé Tchala Vignon Zomahoun, Maxime Sasseville, Marie-Eve Poitras

**Affiliations:** 1 Department of Family Medicine and Emergency Medicine, University of Sherbrooke, Sherbrooke, Canada; 2 Department of Family Medicine, McGill University, Montreal, Canada; 3 School of Physical and Occupational Therapy, McGill University, Montreal, Canada; 4 Centre of Excellence for Partnership with Patients and the Public (CEPPP), CHUM Research Center, Montreal, Canada; 5 Ingram School of Nursing, McGill University, Montreal, Canada; 6 Department of Social and Preventive Medicine, Laval University, Quebec, Canada; 7 School of Rehabilitation, University of Montreal, Montreal, Canada; 8 Department of Management, Evaluation, and Health Policy, School of Public Health, University of Montreal, Montreal, Canada; 9 Faculty of Nursing Sciences, Laval University, Quebec, Canada

**Keywords:** Patient-reported outcome measures, Patient-reported experience measure, PREMs, PROMs, Healthcare professional organizations, Patient organizations, Environmental scan, Learning health system, Interviews

## Abstract

**Background:**

Patient-reported outcome measures (PROMs) and patient-reported experience measures (PREMs) are becoming essential parts of a learning health system, and using these measures is a promising approach for value-based healthcare. However, evidence regarding healthcare professional and patient organizations’ knowledge, use and perception of PROMs and PREMs is lacking.

**Objectives:**

The objectives of the study were to: 1- Describe the current knowledge and use of PROMs and PREMs by healthcare professional and patient organizations, 2- Describe the determinants of PROMs and PREMs implementation according to healthcare professional and patient organizations.

**Methods:**

We conducted an environmental scan using semi-structured interviews with representatives from healthcare professional and patient organizations. Interviews were recorded and live coded based on the Franklin framework. We used inductive and deductive thematic analysis to extract information about the main themes addressed during the interview (awareness of PROMs and PREMs, examples of implementation and use of PROMs and PREMs, tools used, vision for future implementation, barriers and facilitators to implementation and the best way to collect PROMs and PREMs data).

**Results:**

63% of healthcare professional organizations (*n* = 19) and 41% of patient organizations (*n* = 9) that were contacted agreed to have a representative interviewed. The representatives from both the healthcare professional and patient organizations acknowledged the importance of assessing patients’ experience and outcomes. However, they considered the implementation of PROMs and PREMs tools to be scarce within their organizations, in clinical practice and in the education system. Patient organizations were worried that overuse of PROMs and PREMs could lead to depersonalization of practice. Barriers to implementing PROMs and PREMs included lack of awareness of tools, resistance to change and lack of motivation to complete or explain the questionnaire. Barriers also included factors such as lack of financial, technological and human resources and issues with integration of data and inconsistency of digital platforms.

**Conclusions:**

This environmental scan revealed a lack of awareness of tools by healthcare professional and patient organizations’ representatives and limited implementation. Adequate training, technological integration, and demonstration of PROMs and PREMs benefits to foster broader adoption in clinical and organizational settings is dearly needed. Addressing these challenges is essential for enhancing value-based care.

**Supplementary Information:**

The online version contains supplementary material available at 10.1186/s41687-024-00795-9.

## Background

Value-based healthcare, an approach centered on patients’ needs in which the patient is directly involved in decisions regarding his health condition and these decisions are then acted upon, provides healthcare adapted to its expectation as opposed to outdated models of care involving a more paternalistic approach [1–3]. In this context, assessing patients healthcare outcomes and experience is becoming increasingly common in the current healthcare system [4–6]. Indeed, patients are considered experts of their condition, and considering their feedback is crucial for enhancing healthcare quality [7]. Patient-reported outcome measures (PROMs) and patient-reported experience measures (PREMs) are validated self-reported tools that provide a standard way to evaluate patients’ outcomes and experience without any third-party interpretation [3]. A key driver for their implementation in clinical settings is that PREMs and PROMs can improve patients and professionals’ interactions and emphasize shared decision-making [8]. PREMs also allow for assessing the patients’ experience with care, whereas PROMs give an insight into the patient’s perception of their health, symptoms and well-being [9].

PROMs allow for a contextualized understanding of patients’ symptoms [10, 11]. Since PROMs provide patients with a vocabulary that can be used to describe their symptoms further, it improves patient’s self-management and communication with their providers [11–13]. PROMs also facilitate interprofessional communication by standardizing patients’ health outcomes and improves early detection of diseases and exacerbations [12–15]. PREMs are helpful in the identification of clinical or healthcare organizations’ weaknesses aiming at improving patients’ experience of care [16]. A proportional relationship exists between positive patients’ experiences, especially regarding trust in professional staff and communication levels, and their health outcomes [17].

Despite all these recognized benefits of using PROMs and PREMs, implementation in usual care is still scarce [18, 19]. Researchers identified multiple barriers at the patient, healthcare professional and organizational levels, explaining low implementation levels of PROMs and PREMs in clinical settings and within organizations [9, 20, 21]. These barriers include patients’ lack of familiarity with electronic devices, lack of healthcare professionals’ time and inadequate information technology for using and analyzing PROMs and PREMs [20]. Thus, using these tools is still not common in clinical practice, which indicates a need for adjustments to maximize the efficiency of its use [9, 12, 22]. Although the barriers are well documented, evidence regarding the point of view of PROMs and PREMs of associations or professional orders governing the practices of healthcare professional organizations (i.e., associations or professional orders governing the practices of healthcare professionals in Quebec) and patient organizations (i.e., representing and advocating the rights and interests of patients) is limited [9]. We will refer to these organizations as healthcare professional and patient organizations. Their point of view is essential to propose relevant and efficient solutions to facilitate the implementation of PROMs and PREMs and move towards a value-based and learning health system based on the adoption of innovative practices [23].

The aim of our study was to understand the current implementation of PROMs and PREMs in Quebec, Canada. The specific objectives were: (1) to describe the current knowledge and use of PROMs and PREMs by healthcare professional and patient organizations in Quebec, Canada, and (2) to describe the determinants of PROMs and PREMs implementation according to healthcare professional and patient organizations.

## Methods

**Fig. 1:**
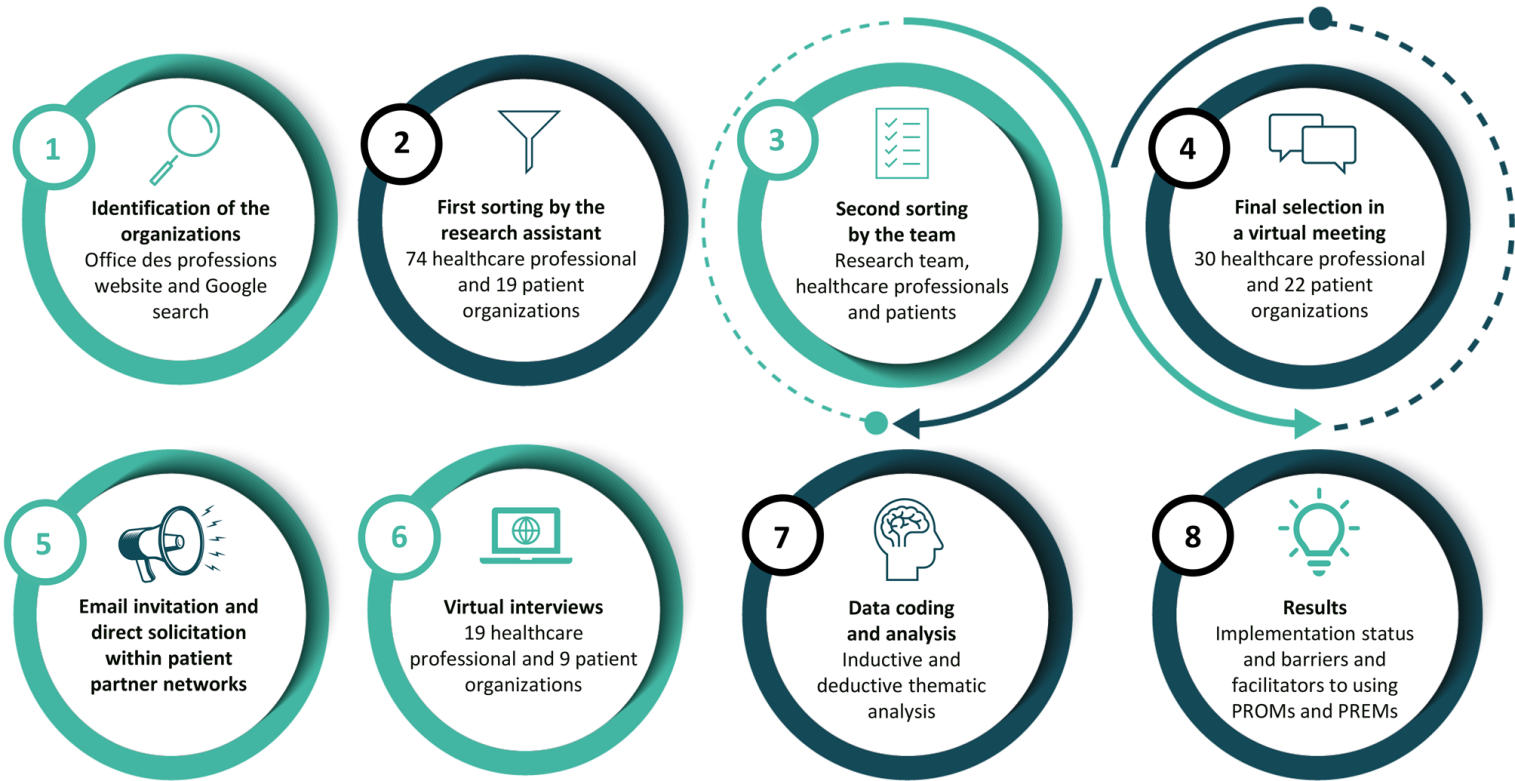
Schematization of the method of the project

We conducted an environmental scan [24, 25] to understand the current implementation of PROMs and PREMs in Quebec, Canada. In our project, this method allowed us to use an agile and pragmatic approach for questioning and understanding healthcare professional and patient organizations’ representatives to support the answer to our aim. This approach has been acknowledged as a crucial and effective public health tool, providing valuable insights to inform policy, planning, and program development within an organization [24–26]. Environmental scans have been used in healthcare using interviews with stakeholders to examine the current state of program implementation and guide quality improvement initiatives [24, 27]. Similarly, in the context of this project, we performed semi-structured interviews to explore the point of view of Quebec healthcare professionals and patients’ organizations regarding the use of PROMs and PREMs in healthcare. The schematization of the different steps of the method of this project is presented in Fig. 1.

### Sampling and recruitment

In the fall of 2021, a research assistant (PB) conducted an Internet search to identify healthcare professional and patient organizations. We identified professional orders related to the health and social services systems on the *Office des professions du Quebec*, a governmental organization that regulates professional practices, website. We also searched the first five pages on Google to identify professionals’ associations that are not professional orders. We searched patient organizations using Google (first five pages) and we selected organizations representing patients living with the most common health problems in Quebec (i.e., arthritis, cancer, chronic respiratory diseases, dementia, diabetes, cardiovascular diseases, mental illness, neurological conditions, and osteoporosis). (31) A patient partner from our team also suggested three patient partners of different organizations that could share their experience with PROMs and PREMs. Our search identified 74 healthcare professional organizations and 19 patient organizations. A research assistant (PB) selected the leading patient and professional organizations in Quebec susceptible to implement PROMs and PREMs. The list was emailed to the team members (patient partners, researchers, research assistants, clinicians) for validation and they were asked to select organizations to contact. We based our selection on the variability and heterogeneity of the organizations representing healthcare professionals and patients. We held two virtual meetings with the team members on Microsoft Teams to discuss which organizations to include and make the final selection of those to be contacted. A research assistant (PB) sent an initial email to the address provided on the organization’s website, usually a director or president, or using a generic email address. A reminder was sent ten days later. The research assistant called the organization if we did not receive a response, and a phone number was available. If a person agreed to participate, a telephone or Microsoft Teams virtual interview was scheduled, according to the respondent’s availability.

### Data collection

We co-constructed two semi-structured interview guides (Supplementary material 1) with the interdisciplinary expertise of the research team and guided by the Franklin framework to Guide the Collection and use of PROMs in the learning healthcare system [28]. The Franklin framework was designed to guide the implementation of PROMs. In our study, it was used in developing our semi-structured interview guide to question the representatives on how PROMs and PREMs are implemented in healthcare professional and patient organizations. The major themes addressed in the interview guides were current practices regarding the use of PROMs and PREMs, tools used for measurement, potential benefits, challenges, and future visions for implementing PROMs and PREMs into various organizational aspects such as professional development and clinical decision-making processes. The guide was pre-tested during the first two interviews. Interviews were conducted from November 2021 to February 2022. The principal researcher (MEPo) listened to the interviews and adapted the content of the guide with the interviewer for subsequent interviews. Four research team members (PB, CC, JB, AM) conducted semi-structured recorded virtual or telephone interviews to discuss the use of PROMs and PREMs in professional. During the interview, each interviewer produced a summary sheet with the representative role, main ideas emerging from the interviews and memorable some citations.

### Data analysis

An Excel spreadsheet (Supplementary material 2) was developed based on the Franklin framework [28]. A research assistant (PB) used an inductive and deductive thematic analysis approach for coding the recorded interviews and generating themes in the Excel spreadsheet using a live coding method (i.e., while listening to them) [29, 30]. Live coding is an alternative to coding transcripts that can be beneficial for preserving the voice and specific meaning of the words used by the participant, since listening to the interview preserve the context and non-verbal behaviour [29]. A second member (CC) of the team listened to all the interviews and validated the coding and themes in the Excel spreadsheet. The principal investigator then reread the entire coding and themes with both coders to ensure a common understanding and the accuracy of the thematic analysis. According to the Tri-Council Policy Statement for human ethical research, informants were not considered as research participants, and we were exempted of ethic approval by the ethic research committee.

## Results

**Fig. 2:**
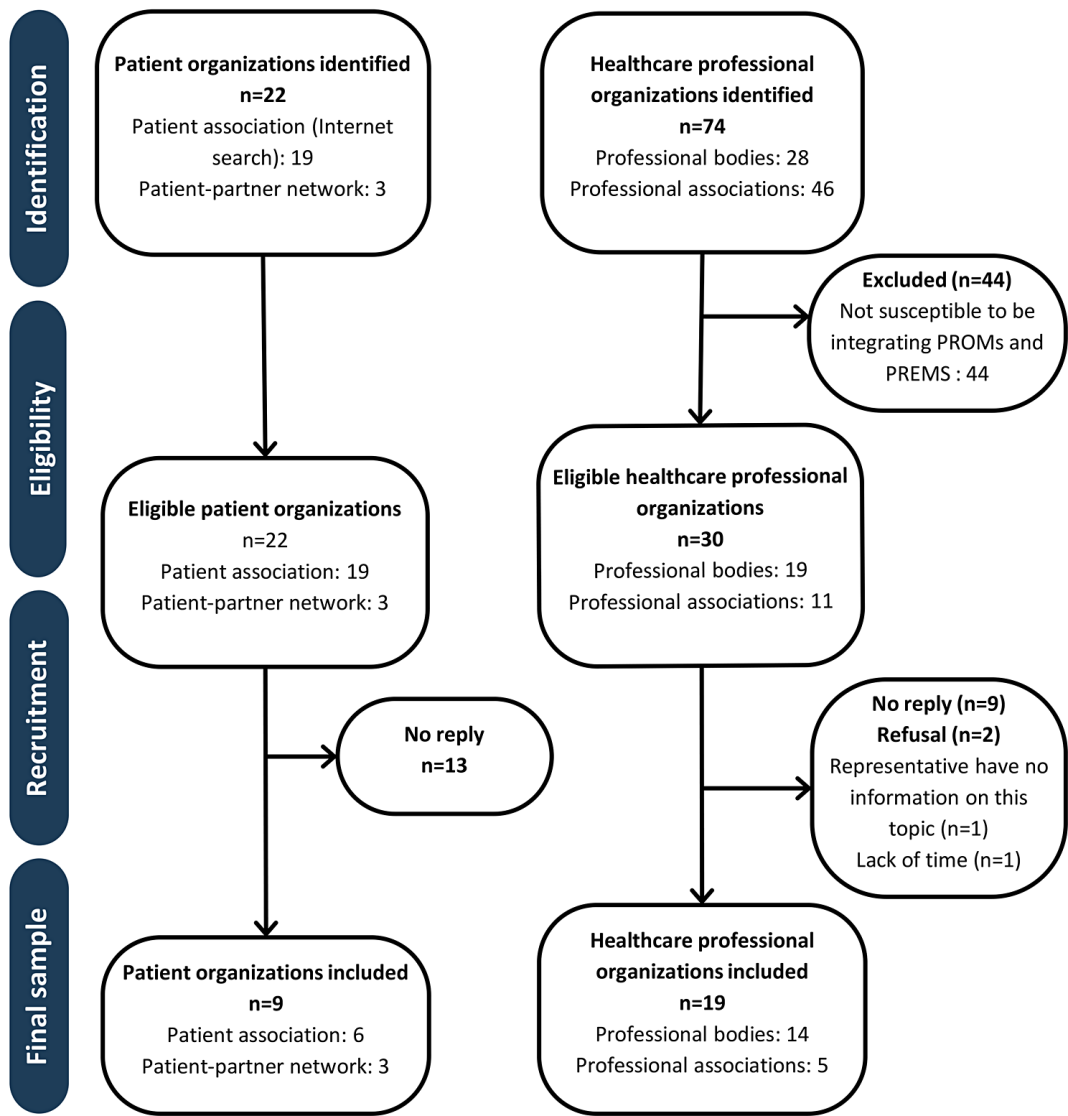
Flow diagram of healthcare and patient organizations’ selection process

Thirty healthcare professional organizations were contacted for an interview, of which nine did not reply, and two refused to participate. Twenty-two patients’ organizations were contacted for an interview, and 13 did not reply to our invitation. We conducted 28 semi-structured interviews, 19 with healthcare professional organizations and nine with patients’ organizations or patient partners. Thus, 63% of healthcare professional organizations and 41% of patients’ organizations responded positively to our invitation. Figure 2 presents the enrollment process for healthcare professional and patient organizations. Healthcare professional and patient organizations that were interviewed are presented in Table 1.

**Table 1:** Healthcare professional and patient organizations interviewed

Healthcare professional organizations	Patient organizations
• Association of Senior Executives in Health and Social Services • Quebec Association of Orthotists Prosthetists • Quebec Association of Pharmacists in Healthcare Institutions • Quebec Association of Specialized Nurse Practitioners • Quebec College of Physicians (professional order) • Quebec Federation of Kinesiologists • Quebec Professional Order of Auxiliary Nurses • Quebec Professional Order of Chiropractors • Quebec Professional Order of Dental Hygienists • Quebec Professional Order of Dentists • Quebec Professional Order of Educational Psychologists • Quebec Professional Order of Midwives • Quebec Professional Order of Nurses • Quebec Professional Order of Occupational Therapists • Quebec Professional Order of Pharmacists • Quebec Professional Order of Physiotherapists • Quebec Professional Order of Podiatrists • Quebec Professional Order of Respiratory Therapists • Quebec Professional Order of Speech-Therapists and Audiologists	• Canadian Cancer Society • Patient partner 1 • Patient partner 2 • Patient partner 3 • Quebec Cancer Priority Coalition • Quebec Chronic Pain Association • Quebec Coalition for Orphan Diseases • Diabetes Quebec • Weight Coalition

Interviews lasted between 20 and 40 min. We present the perspective of healthcare professional and patient organizations on the implementation of PROMs and PREMs in various settings, and determinants of the implementation of PROMs and PREMs in clinical settings from each perspective and relevant citations.

### Healthcare professional organizations’ perspective

#### Awareness of PROMs and PREMs in healthcare professional organizations

Healthcare professional organizations’ representatives responded that the concept of self-reported measure was familiar but were mostly not aware of PROMs and PREMs tools.

The more formalized and standardized self-reported measures, yes, that is new. (PRO-010)

Representatives reported consulting patients regularly within their organization but did not report using PROMs or PREMs to validate interventions’ impact or enhance professional practice. However, some organizations expressed interest and willingness to use PROMs and PREMs in the future, as one representative mentioned:

I will definitely talk to my colleague who is responsible for inspection; we can improve [our practice]; we can improve. (PRO-010)

#### Implementation of PROMs and PREMs in clinical practice

Healthcare professional organizations’ representatives were not aware of PROMs and PREMs use in the clinical practice of their members. However, all the organization representatives that were interviewed reported that clinicians are incorporating the patients’ perception of their outcomes in their interventions by asking patients about their perception of their health (mostly orally and sometimes with questionnaires). According to respondents, the patients’ perception is often documented in clinical records.

In the history, there are questions about pain (discomfort, intensity 1–10, type of pain, unpleasantness 1–10, etc.) (PRO-017)

[Professionals] question, observe, and report what patients mention about their health outcomes in observation notes (PRO-05)

However, incorporating formal PROMs and PREMs was not a commonly adopted practice by healthcare professionals that are members of their organization.

We know it exists, but it’s not a practice that is adopted by [category of professionals represented by the representative interviewed]. (PRO-07)

According to the respondents, PREMs need to be implemented in clinical practice. Some healthcare professionals use in-house non-validated questionnaires to measure patient satisfaction with services received and the overall care experience.

There are always satisfaction surveys; it’s part of the [professional] job to adjust their work plan. The patient experience is something that is embedded in the practice. […] They are not validated surveys; we use in-house surveys. (PRO-018)

Some representatives mentioned that healthcare professionals verbally ask people who receive their services about their satisfaction, as they prefer to discuss rather than administer a questionnaire and then analyze the responses. In contrast, others indicated that patients spontaneously express their satisfaction with the services they receive.

Needs assessment is done in various contexts through initial data collection. The patient experience is reported directly by the patient or by the professional. It is more informal; there is no questionnaire. (PRO-010)

Healthcare professional organization representatives mentioned that information regarding PROMs and PREMs needs to be shared with their members and that there is a need to integrate more patients’ point of view into clinical practice, as this participant reported:

We have to stop being paternalistic. Patients are increasingly educated and knowledgeable about therapy, they have questions, requests […] we have to change our attitude as professionals, we can no longer have the “I know what’s good for you [attitude]. (PRO-015)

#### Implementation of PROMs and PREMs into undergraduate training and continuing professional development

Only one of the professional organizations’ representatives reported that using PROMs through validated questionnaires was taught in the undergraduate training of healthcare professionals.

At the university, it is mandatory to complete the PROMs questionnaires at each visit […] it gets lost quickly though because of time constraints. (PRO-016)

Some healthcare organization representatives reported that discussion around patients’ point of view during undergraduate training and continuous education is limited to the patient partnership.

The organizations’ representative reported that the patients’ perception, although rarely included in continuing professional development, is an important concept to discuss.

Improving the skills […] must be done through initial training. Initial training is not suited to this approach [patient perception]. […] It would be interesting to develop training or clinical practice guides. (PRO-012)

In conclusion, healthcare professional organizations’ representatives were not aware of PROMs and PREMs tools, but they believe that their organization should use these tools. Regarding PROMs and PREMs implementation in clinical practice, most professional organizations’ representatives mentioned that healthcare professionals’ members of their organization are probably not using standardized PROMs and PREMs but consider these tools valuable and necessary in the practice of the healthcare professionals. Healthcare professional organizations’ representatives also mentioned that PROMs and PREMs are also not implemented in professionals’ undergraduate training and continuing professional development.

### Patient organizations’ perspective

#### Awareness of PROMs and PREMs in patients’ organizations

The concept of patient self-reported measures, whether PROMs or PREMs, appeared new to most patient organizations. Representatives indicated that they perceived their role as more related to listening and supporting patients than to data analysis, as reported by one representative:

The primary role of the association is to listen and support our members. Then, to establish the needs and priorities of the members, we organize coffee meetings. (PT-06)

Organizations’ representatives have reported they are using coffee meetings, forums, focus groups, patient panels, summits, or non-validated in-house questionnaires to collect information regarding their members. In general, most organizations feel very unconcerned with PROMs and PREMs. A quote from one of the representatives of patient organizations represent this situation:

I think you’re in your world; you think self-reported measures exist everywhere. Give me an example of how it’s used, because no, it doesn’t. (PT-06)

#### Implementation of PROMs and PREMs in clinical practice

Patient organizations’ representatives believed using PROMs and PREMs could help enhance healthcare professionals’ practices. Patient organizations believed that the recognition of patients’ experiential knowledge needs to be sufficiently valued in treating their condition.

Including more of the patients’ perspective would improve tailored care. (PT-05)

There is not enough valuing of the input and expertise of patients’ experiential knowledge. This is something that [this organization] is actively advocating for. (PT-03)

Many healthcare professionals still observe a paternalistic approach, which results in decreased patient engagement in care centered on the needs of the patients.

It is not usual for professionals to include patients’ perspectives. It’s a more paternalistic approach that we encounter with the majority of professionals. (PT-02)

Including the patient perception is very professional dependent, as mentioned by a representative of a patient’s organization.

Pivot nurses and nurses are great at integrating the patient experience. Specialists are more difficult…On the other hand, young people are often more human and open. What helps is the patient partner offices in hospitals. There is room to enhance the patient partnership approach. However, it’s extremely dependent on the professional. (PT-03)

However, patient organizations feared that the overuse of questionnaires could lead to depersonalization and dehumanization of practice.

I hope that new practices will be used to enhance the quality of care, not to enhance dehumanization and depersonalization of care. (PT-01)

In conclusion, despite current limited use of PROMs and PREMs into patient organizations, representatives are aware that including patients’ experience and needs should be more integrated into their organization and clinical practice. However, they are still skeptical about the necessity and realism of implementing PROMs and PREMs into their organization. They worried that implementing PROMs and PREMs in clinical practice could lead to depersonalization of care.

### Determinants of PROMs and PREMs implementation

According to healthcare professional and patient organizations, several factors could facilitate the implementation of PROMs and PREMs at the clinical and organizational levels. One of the main elements that could help stakeholders implement PROMs and PREMs in clinical settings would be to demonstrate the efficiency of their use in the short and long term. According to healthcare professional organizations’ representatives, it is essential to demonstrate the added value of using these measures to healthcare professionals so they would agree to use them into current practice, as reported by one representative of a healthcare professional organization.

[…] we have to believe in it, we have to take the time to reach it. (PRO-01)

Patients’ organizations emphasized that patients must be informed about the usefulness of these measures to make an informed decision about completing a PROMs or PREMs questionnaire. This would also minimize the perception of completing questionnaires as an additional burden on the patient.

What would be helpful is to have information, not just questions. Patients should have access to information in addition to the questionnaires. We need to know what we’re doing by filling out these questionnaires. Information is a tool. Upstream, we will also need education. We need to be able to self-educate and self-train with our health. All health professionals will have to work together, as well as with the patient. (PT-01)

To facilitate the implementation of these measures into their practice, healthcare professionals would like to have access to adequate training on the available tools, their use and interpretation.

[…] for intermediate theories like self-reported measures, we have been trying to train ourselves for several years, but the problem is that we don’t have a trained teacher to pass that on to us. The older ones must incorporate it and train the others. (PRO-06)

Both healthcare professional and patient organizations addressed issues that might impede the implementation of PROMs and PREMs. Accessibility of the tools and application difficulties, primarily at the technological level, are the main challenges professional and patient organizations report. The lack of integration of PROMs and PREMs into the electronic medical record system or other technological tool accessible to all healthcare professionals and patients presents a major obstacle.

[…] not everyone has access to the computerized record; ideally, everyone should have access, even the patients. (PRO-03)

**Fig. 3:**
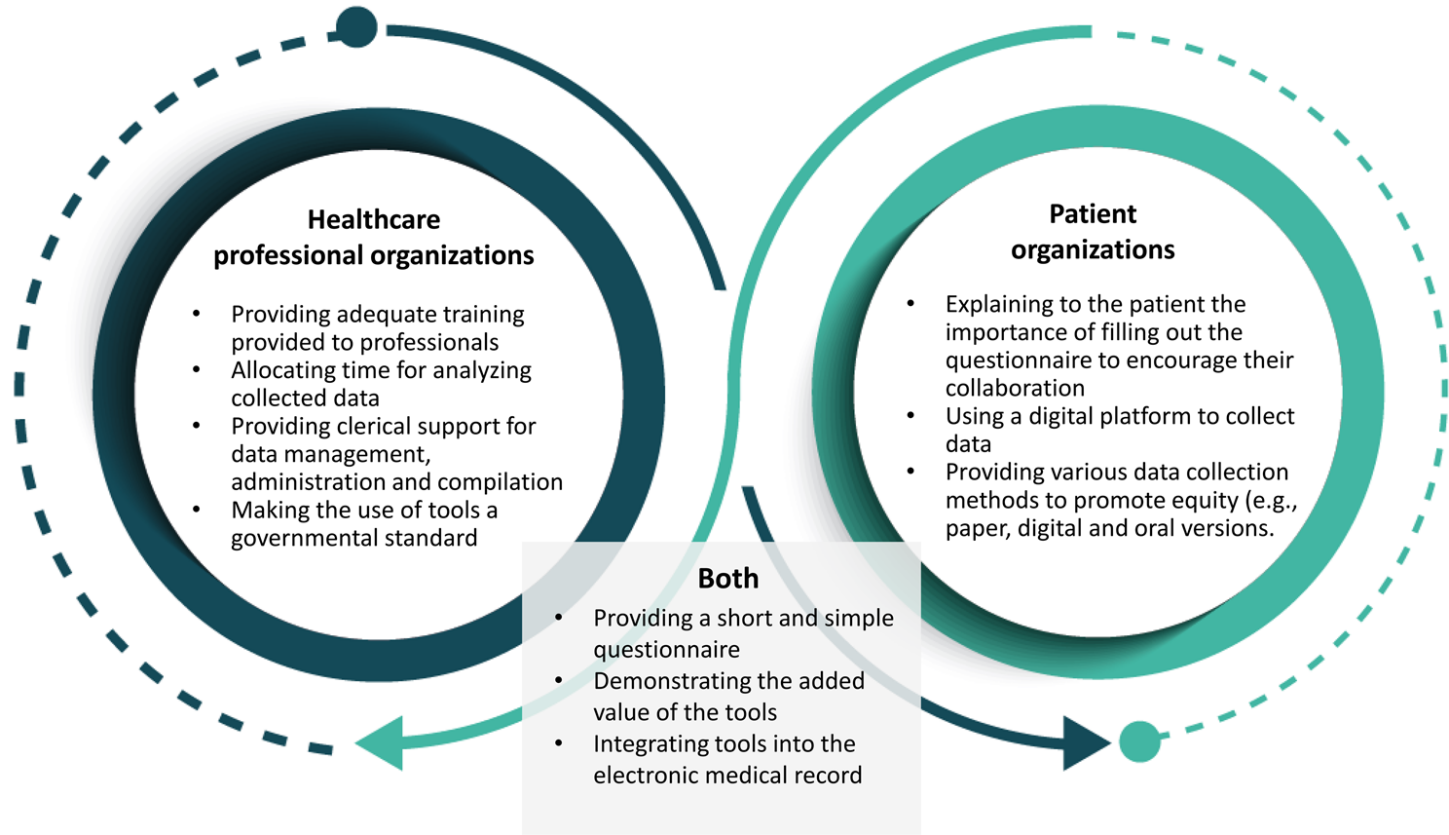
Facilitators to implementing PROMs and PREMs

**Fig. 4:**
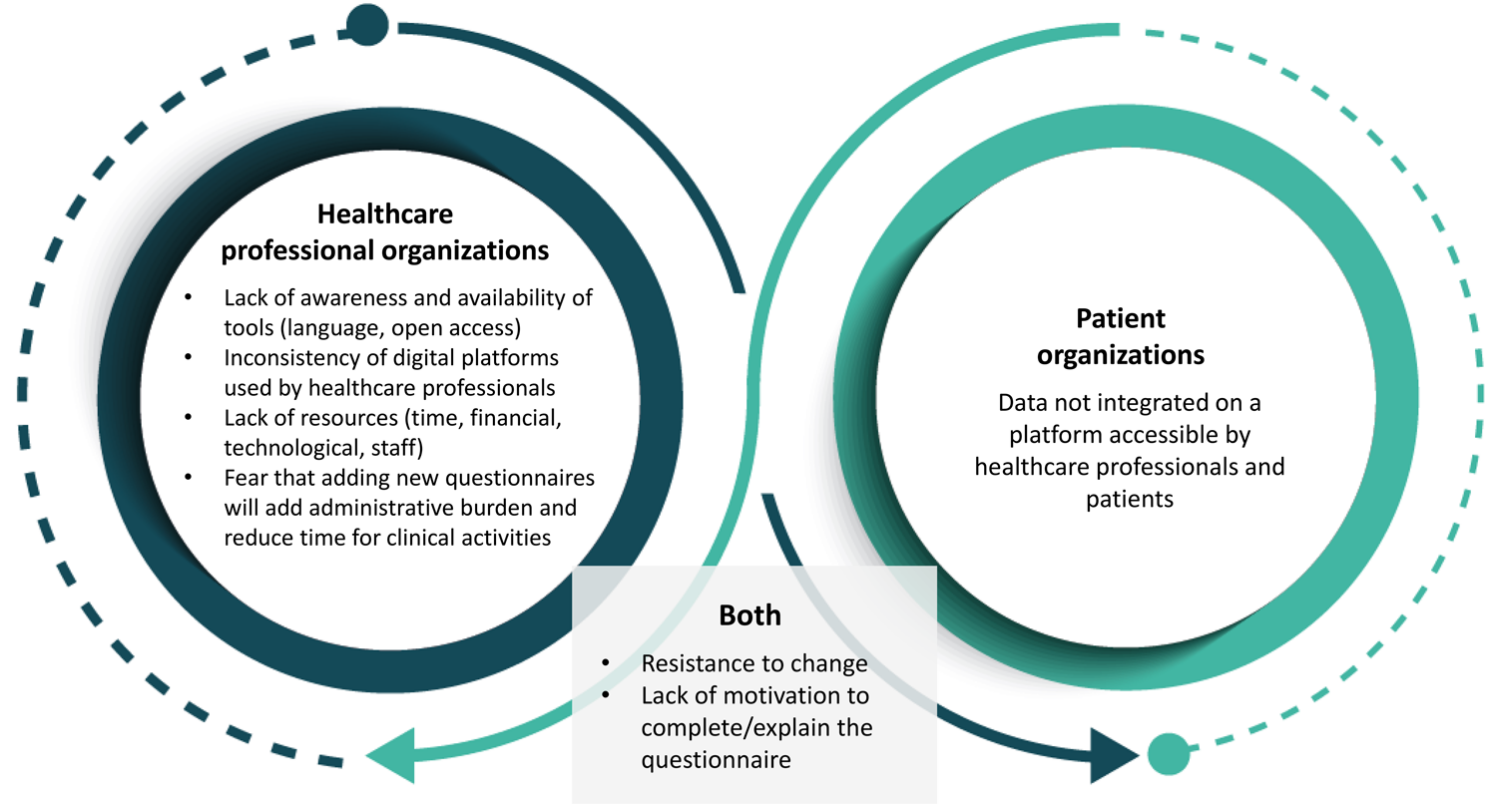
Barriers to implementing PROMs and PREMs

The complete list of barriers and facilitators to implementing PROMs and PREMs is presented in Figs. 3 and 4.

This figure presents the facilitators to implementing PROMs and PREMs according to healthcare professional organizations, patient organizations and facilitators that were mentioned by both type of organizations.

This figure presents the barriers to implementing PROMs and PREMs according to healthcare professional organizations, patient organizations and facilitators that were mentioned by both type of organizations.

## Discussion

In our environmental scan, we performed semi-structured interviews to describe the current use of PROMs and PREMs in healthcare professional and patient organizations and to describe determinants to successful PROMs and PREMs implementation. PROMs and PREMs are essential for improving healthcare systems, as patients’ experience and outcomes are part of the quintuple aim for improving healthcare [31, 32]. Main findings emerging from our study include the lack of awareness and knowledge about using validated PROMs and PREMs in healthcare professional and patient organizations. Barriers at the individual levels that were identified include professionals’ lack of motivation to explain the questionnaire and patients’ disinterest in completing these questionnaires. According to patients’ organizations, it is essential to explain the importance and rationale for filling out the PROMs and PREMs questionnaires to patients. Barriers to implementing PROMs and PREMs include organizational barriers such as lack of time, financial, technological, and human resources to implement the tools routinely.

### Lack of understanding and use of PROMs and PREMs

Despite that most healthcare professional organizations’ representatives that we interviewed acknowledged that patients’ perception is essential, the use of validated tools to measure PROMs and PREMs was lacking. Healthcare professional organizations mentioned that clinicians do not use validated self-reported measures during patient consultations but instead rely on in-house tools or verbal exchanges. However, using only verbal exchanges is limited by social desirability bias from patients towards their healthcare professional [33]. Insufficient information about the usefulness and availability of validated PROMs and PREMs could explain the lack of awareness regarding the necessity of implementing PROMs and PREMs instead of using non-validated tools [3, 34–37]. Standardized instruments should be used since they are developed using validation steps and have available information on psychometric qualities and lead to an accurate picture of a patient’s health status and experience [34]. Nevertheless, it is important to mention that these changes should not be imposed and should respect the readiness level of individuals [38].

An interesting finding from our study is that healthcare professional organizations have mentioned that PROMs seem to be more commonly known and used than PREMs, which is concordant with two recent reviews [39, 40]. This situation could be explained by the larger number of studies conducted regarding PROMs compared to studies regarding PREMs over the last few years, making PROMs potentially more accessible [41].

Most patient organizations’ representatives were not aware of PROMs and PREMs existence. They mentioned that their role is more to support the patients who are members of their organization and not to evaluate and analyze patients’ health status. However, patient organization representatives considered it could be useful for healthcare professionals to use PROMs and PREMs when doing disease management with the patients. Yet, our research team considers that assessing patients who are members of the organization using standardized measures such as PROMs and PREMs could help identify areas within the healthcare system and community that require improvement. Moreover, patient organizations could be involved in developing PROMs and PREMs initiatives as stakeholders since they represent the interest of patients.

### Determinants to PROMs and PREMs implementation

Respondents from healthcare professional and patient organizations have identified many barriers and facilitators to implementing PROMs and PREMs at the healthcare professional and patient level, but also at organizational levels such as in healthcare professionals and patient organizations, and in academic organizations.

We identified barriers at the healthcare professional level, including the lack of awareness of tools and the lack of ability to analyze and interpret data. The organizations’ representatives also fear that using PROMs and PREMs will increase healthcare professionals’ workload and decrease the quality of care provided to patients. This misinterpretation represents a significant challenge in implementing PROMs and PREMs and addressing this challenge should be prioritized. Although a lack of time has been highlighted as an organizational barrier to using PROMs and PREMs, both in the literature and in our project, some studies have reported that using self-reported measures in clinical settings does not take more time and may even save time [12, 42]. Using PROMs and PREMs can eliminate the need to ask patients questions that the tool have already addressed. To address the previously identified barriers, Santana and colleagues emphasize the importance of integrating education and training from the early stages of implementing PROMs and PREMs in healthcare settings [43]. This approach could help healthcare professionals in effectively using the data collected by the questionnaires [43–45].

Another organizational challenge to PROMs and PREMs implementation reported by healthcare professional organizations is the lack of human resources to handle administrative tasks and a lack of funds to implement the tools routinely. A key element highlighted in the literature to decrease the impact on human and financial resources is to promptly establish a transparent and adaptable process for collecting, analyzing, and acting on data generated from PROMs and PREMs [10, 44, 46, 47]. Moreover, designing a process compatible with the values of healthcare professionals and work organization can facilitate the successful implementation of PROMs and PREMs in healthcare settings [44].

Some healthcare professional organizations have mentioned facing obstacles specific to their environment, such as the perception that PROMs and PREMs are less relevant when patients require frequent follow-ups. It is, therefore, crucial to raise awareness among organizations about integrating these tools into their professional practice, while adapting the implementation of PROMs and PREMs to the clinical context [12]. Healthcare professional associations have also reported a lack of access to continuing professional development related to PROMs and PREMs implementation. Several authors have also noted this observation, hence the importance of raising awareness among organizations that offer continuing education to integrate training on these tools [8, 20, 22, 43, 46, 48]. Academic undergraduate and postgraduate organizations should raise awareness among students about PROMs and PREMs and their utility in clinical practice, and provide opportunities to learn how to implement these tools (practical work, use in electronic health records).

The representatives of patients’ organizations that were interviewed emphasized that patients should be informed about the usefulness of completing the questionnaires to encourage their participation. Indeed, as reported in the literature, although the majority of patients are in favour of completing PROMs and PREMs, some may doubt the relevance of these tools or of some questions for improving their care due to a lack of clear explanation from their healthcare professional [12, 20]. Furthermore, patients may disengage from filling the questionnaire if they perceive that healthcare professionals do not take the time to discuss the results with them [49].

Developing skills focused on implementing PROMs and PREMs supports an optimal care trajectory in line with the needs of patients. The proposed recommendations should be applied not only in clinical settings but also in the structure of healthcare professional and patient organizations and in the government, by making the use of PROMs and PREMs a government standard. The government can support clinicians in implementing PROMs and PREMs implementation, as this organization promote the adoption of innovative practices and appropriate health behaviours [36].

### Strengths and limitations

This environmental scan provides a comprehensive snapshot of the current landscape surrounding the implementation of PROMs and PREMs in healthcare, with its strength lying in the ability to capture a wide array of points of view by interviewing diverse healthcare professional and patient organizations. The study ensures the relevance and authenticity of its findings by obtaining real-world insights directly from healthcare professional and patient organizations. Identifying determinants and opportunities through the environmental scan will help the healthcare community with valuable insights to inform future strategies to optimize the implementation of PROMs and PREMs. While the environmental scan method provides a rich understanding, it is not without limitations. Interviewees may have been influenced by social desirability or individual perceptions. This study being focused on specific healthcare professional and patient organizations in the Province of Quebec in Canada may limit the generalizability of our findings. However, our study provides a detailed understanding of the implementation of PROMs and PREMs in this context and results may be transposable to similar jurisdictional or national contexts. Another limitation of our study is that it was conducted in 2021–2022. The dynamic nature of healthcare practices also poses a challenge, as the rapidly evolving landscape may impact the relevance of findings over time. However, according to the latest projects conducted by our teams related to the use of PROMs and PREMs, despite the healthcare professionals organizations’ intentions to implement these tools, their use remains limited by the barriers mentioned in our manuscript. Despite these limitations, our study allows to lay a foundation for future research and interventions.

## Conclusion

While including patients’ perceptions is recognized as part of best practices for most healthcare professional organizations, the main observation from this environmental scan is that the implementation of PROMs and PREMs into everyday practice by healthcare professionals and patients is still in its early stages. There is a lack of awareness and understanding of PROMs and PREMs optimal and scientifically recognized use. Indeed, healthcare professionals are often insufficiently informed about PROMs and PREMs. Education and promotion of PROMs and PREMs are needed to foster their effective use and enhance professional practice. Importantly, these changes should respect each professional readiness level. Organizations should identify improvement opportunities, evaluate options, prepare for implementation, then proceed accordingly. Supporting organizations at their readiness level and guiding them through the implementation process is crucial for successful integration.

## Electronic supplementary material

Below is the link to the electronic supplementary material.

Supplementary Material 1

Supplementary Material 2

## Data Availability

The data supporting this study’s findings are available from Marie-Eve Poitras. Still, restrictions apply to the availability of these data, which were used under license for the current study and are not publicly available. Data are however available from the authors upon reasonable request and with permission of Marie-Eve Poitras.

## Abbreviations

PROMs: Patient-reported outcome measures
PREMs: Patient-reported experience measures
