# Supplementary material for: How patient-reported outcomes and experience measures (PROMs and PREMs) are implemented in healthcare professional and patient organizations? An environmental scan
Source: J Patient Rep Outcomes. 2024 Nov 15;8:133. doi: 10.1186/s41687-024-00795-9 (PMC11568099; doi:10.1186/s41687-024-00795-9)
Supplement: Supplementary file 1 — Supplementary Material 1 [file 41687_2024_795_MOESM1_ESM.docx]

**Interview guide for healthcare professional organizations**

**Description of the actual situation**

1. First, could you give me a concrete example of what is done by ________________________ (NAME THE PROFESSIONAL) to integrate:
   1. the patients' point of view of their healthcare experience? (e.g., satisfaction with care, was the patient satisfied with the encounter, did they feel listened to? patient-centered care, shared decision-making, was the patient involved in decision-making regarding their health?)
   2. The patient's point of view in relation to the perception of his health? (e.g., perception of health such as well-being, quality of life, symptoms such as pain, fatigue, physical and social functioning, etc.)
   3. Is the concept of measurement self-reported by patients (healthcare experience and health) something new for your organization?
2. How does ___________ (NAME THE ORGANIZATION) integrate the patients’ point of view related to their healthcare experience into the activities carried out with professionals?
3. How ___________ (NAME THE ORGANIZATION) integrates the patients’ point of view related to their health into the activities carried out with professionals?
4. What tools do you use to measure whether patients' point of view of their health or healthcare experience are included in your activities? (e.g., self-reported validated questionnaire completed by the patient)
5. How could tools that measure patients' point of view related to their healthcare experience and health be useful to support the work of ___________ (NAME THE PROFESSIONAL)?
6. How could tools that measure patients' perspectives on their health or healthcare experience be useful in improving the quality of care for the ___________ (NAMING PROFESSIONALS) you represent?
7. How do _________________ (NAME THE PROFESSIONAL) use data from self-reported measures of healthcare experience in their routine practice?
8. How do __________________ (NAME professionals) apply self-reported measures in their daily practice?
9. What is your medium/long-term vision for including information from patient self-reported health measures or self-reported healthcare experience in:
   1. Continuing education?
      1. Do you currently offer continuing education specifically related to patients’ perspectives (experience and perception of health)?
   2. Your decision-making process?
   3. The inspection of the members?
   4. Certification (Examination of the professional orders/associations)
10. Can you tell me about strategies for integrating patient self-reported health measures or healthcare experience into your members' inspection process?
    1. Is this a priority for you?
    2. Describe this priority on a scale of 1 to 5, of which 1 is a low priority and 5 is a high priority.
    3. What is this strategy/What could this strategy be?
11. In your opinion, what would be the elements that could facilitate the integration of self-reported health measures or healthcare experience by patients?
12. What do you think are the elements that prevent or make it difficult to integrate self-reported health measures or healthcare experiences by patients?
13. This concludes our interview. Do you have anything else to add or explain?

*Note to interviewer: Indicate any observations, remarks or thoughts about the interview that you consider relevant in the summary sheet*

**Interview guide for patient organizations**

**Description of the current state of affairs**

1. First, could you give me a concrete example of what is done in ________________________ (NAME THE PATHOLOGY, E.G. PERSON WITH CHRONIC RD) to integrate:
   1. (e.g., satisfaction with care, was the patient satisfied with the encounter, did they feel listened to? patient-centered care, shared decision-making, was the patient involved in decision-making regarding their health?)
   2. The patient's point of view in relation to the perception of his health? (e.g., perception of health such as well-being, quality of life, symptoms such as pain, fatigue, physical and social functioning, etc.)
   3. Is the concept of measurement self-reported by patients (healthcare experience and health) something new for your organization?
2. With the help of measurement tools, how could the patients point of view of the healthcare experience they received be useful in enhancing the professional practice of clinicians who support the patients you represent?
3. With the help of measurement tools, how could the patients point of view of health be useful in enhancing the professional practice of the clinicians who support the patients you represent?
4. How do you think health care professionals integrate the patients perspective on their healthcare experience into their day-to-day practice?
5. In your opinion, how do healthcare professionals integrate the patients’ point of view of their health into their daily practice?
6. If measurement tools were available to report on patients' point of view of their health or healthcare experience, how could your organization use them?

**Areas for improvement**

1. From your perspective, how could healthcare professionals use patient self-reported measures in their practice to better support shared decision-making and patient-centered care?
2. In your opinion, what would be the best way to collect patients' point of view in relation to their healthcare experience and their health in the clinic?
3. This concludes our interview. Do you have anything else to add or explain?

*Note to interviewer: Indicate any observations, remarks or thoughts about the interview that you consider relevant in the summary sheet*
